# Supplementary material for: Cannabidiol inhibits Nav channels through two distinct binding sites
Source: Nat Commun. 2023 Jun 17;14:3613. doi: 10.1038/s41467-023-39307-6 (PMC10276812; doi:10.1038/s41467-023-39307-6)
Supplement: Supplementary file 1 — Supplementary Information [file 41467_2023_39307_MOESM1_ESM.pdf]

Supplementary information

Cannabidiol inhibits Na_v channels through two distinct binding sites

Jian Huang[1,4], Xiao Fan[1,4], Xueqin Jin[2], Sooyeon Jo[3], Hanxiong Bear Zhang[3], Akie Fujita[3], Bruce P. Bean[3,5] and Nieng Yan[1,2,5]

[1]Department of Molecular Biology, Princeton University, Princeton, NJ 08544, USA

[2]Beijing Frontier Research Center for Biological Structures, State Key Laboratory of Membrane Biology, Tsinghua-Peking Joint Center for Life Sciences, School of Life Sciences, Tsinghua University, Beijing 100084, China

[3]Department of Neurobiology, Harvard Medical School, 220 Longwood Avenue, Boston, MA 02115, USA

[4]These authors contribute equally: Jian Huang, Xiao Fan.

[5]To whom correspondence should be addressed: N. Yan (nyan@princeton.edu); B.P. Bean (bruce_bean@hms.harvard.edu).

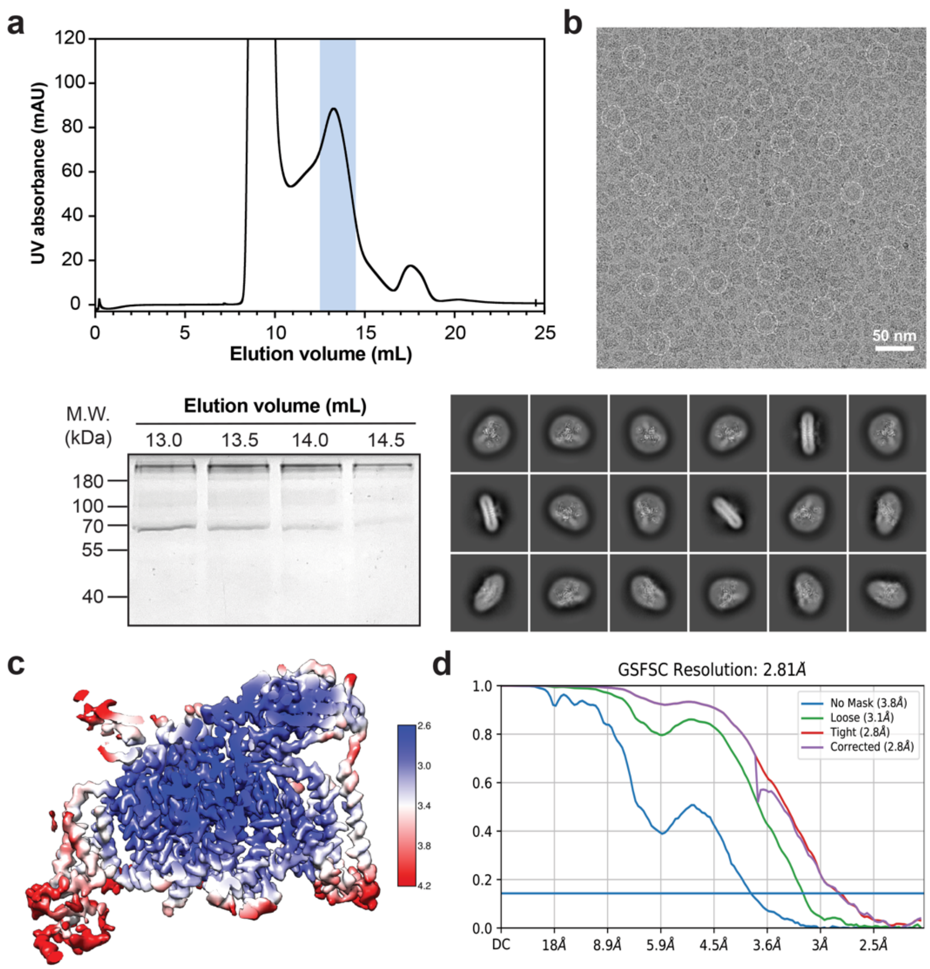

Supplementary Fig. 1 | Structural determination of the human Na$_v$1.7-CBD complex. a The last step purification of the human Na$_v$1.7-CBD complex. Shown here is a representative chromatogram of gel filtration purification. The indicated fractions were resolved on SDS-PAGE and visualized by coomassie blue staining. **b** A representative cryo-EM micrograph (*up*) and 2D classifications (*down*) of the Na$_v$1.7-CBD complex. White circles indicate representative particles in distinct orientations. **c** Local resolution map for the 3D EM reconstitution of Na$_v$1.7 in the presence of CBD. Local resolutions were estimated with CryoSPARC. **d** Gold-standard Fourier Shell Correlation (GSFSC) curves for the overall 3D reconstructions of Na$_v$1.7-CBD complex.

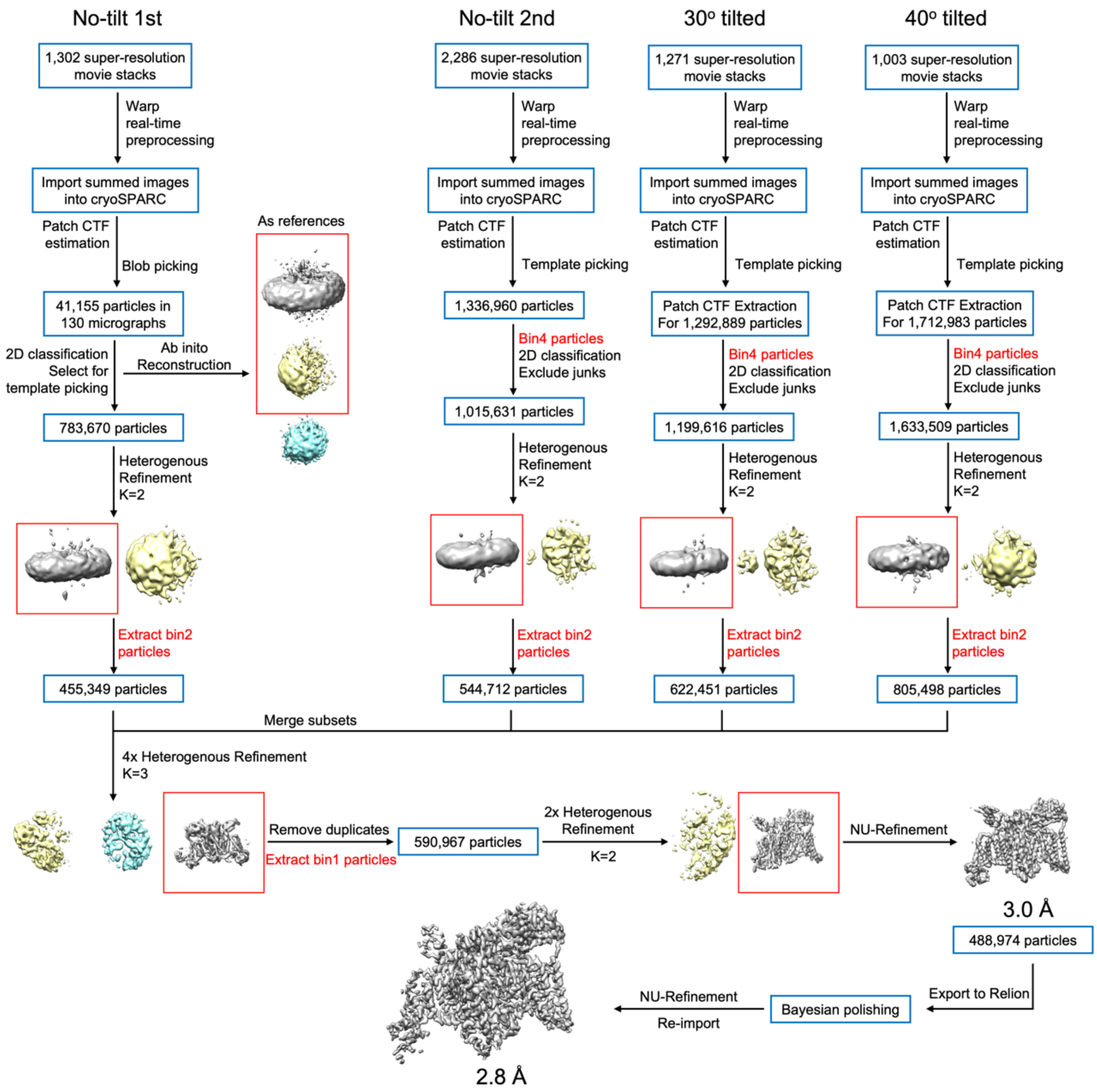

Supplementary Fig. 2 | Flowchart for cryo-EM data processing. Details can be found in Methods.

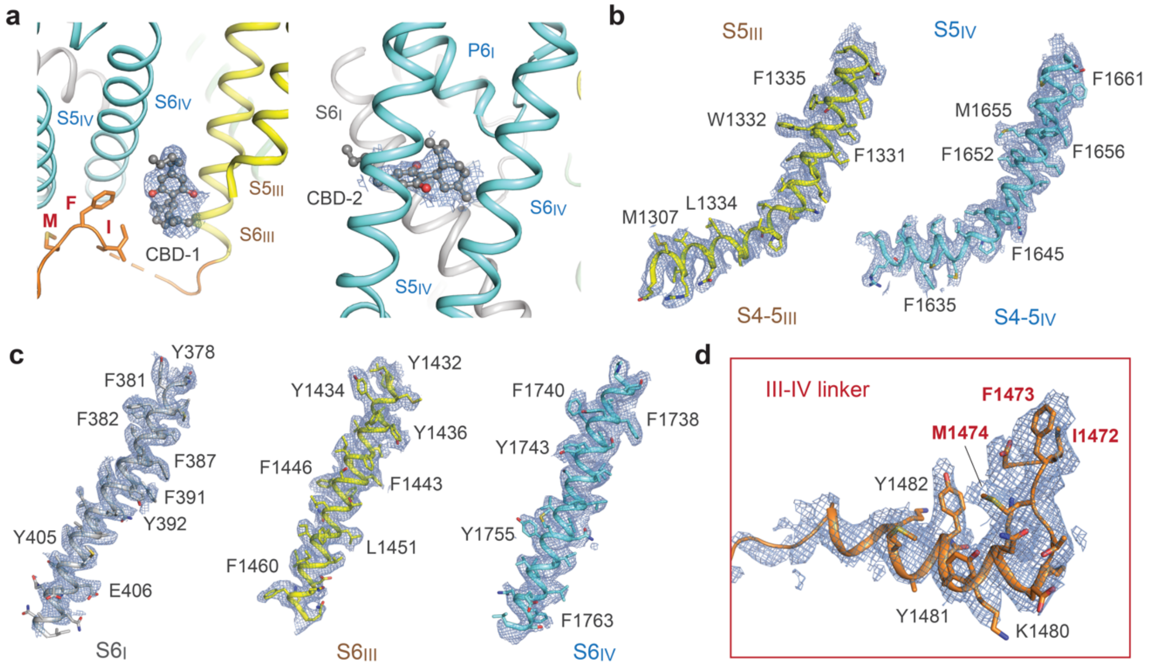

Supplementary Fig. 3 | EM maps for representative segments involved in CBD binding. a Densities for CBD-1 at the I-site (*left*) and CBD-2 at the F-site (*right*). **b,c** EM maps for the pore domain segments that contribute to CBD binding. Distinctive bulky residues are labeled. **d** Densities for the III-IV linker. All the presented local densities are shown as marine meshes contoured at 5 σ level within PyMOL.

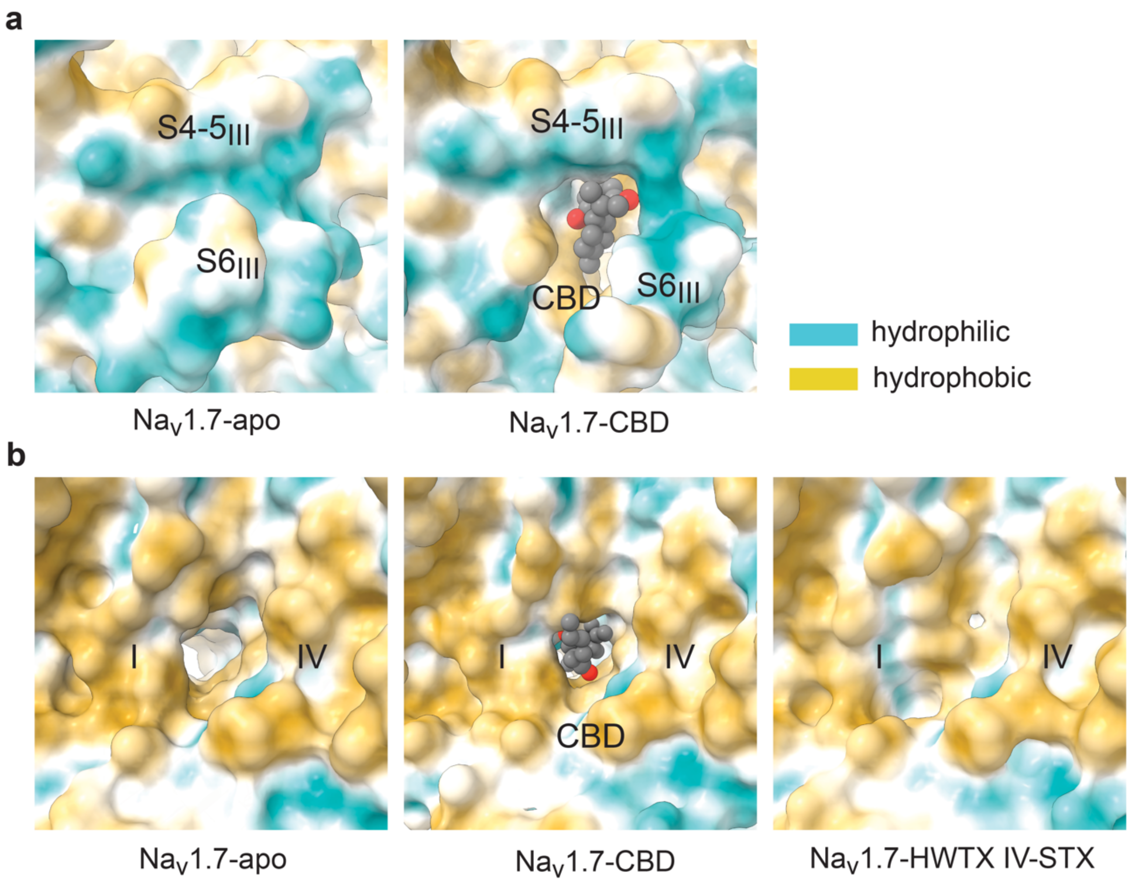

Supplementary Fig. 4 | Hydrophobic environment of the I-site and the F-site. a Conformational changes upon CBD binding at the I-site. Surrounding environment is shown as the hydrophobic surface, calculated in ChimeraX. **b** Different states of the IV-I fenestration in the presence of different ligands. The IV-I fenestration, present in the apo-state (PDB: 7W9K) or CBD-bound channel, is absent in the presence of HWTX IV and STX (PDB: 6J8G).

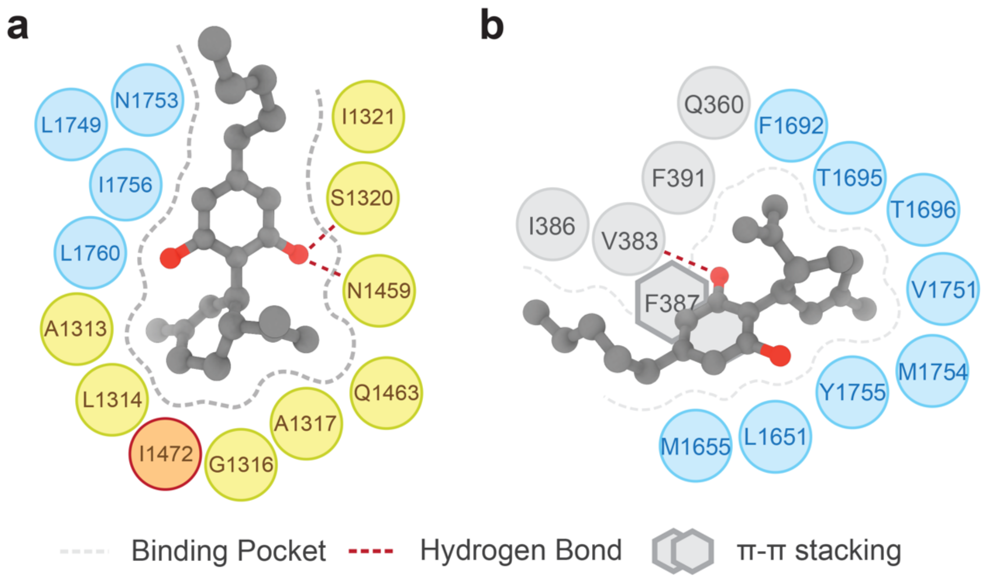

Supplementary Fig. 5 | Plane diagram of residues constituting the I-site and the F-site. The residues constituting the I-site are shown within a 4-Å cutoff distance from CBD (**a**) and 5 Å cutoff for the F-site (**b**). The binding pocket and potential H-bonds are indicated by gray dashed contour and red dashed lines. The residue involved in π-π stacking is shown in hexagon.

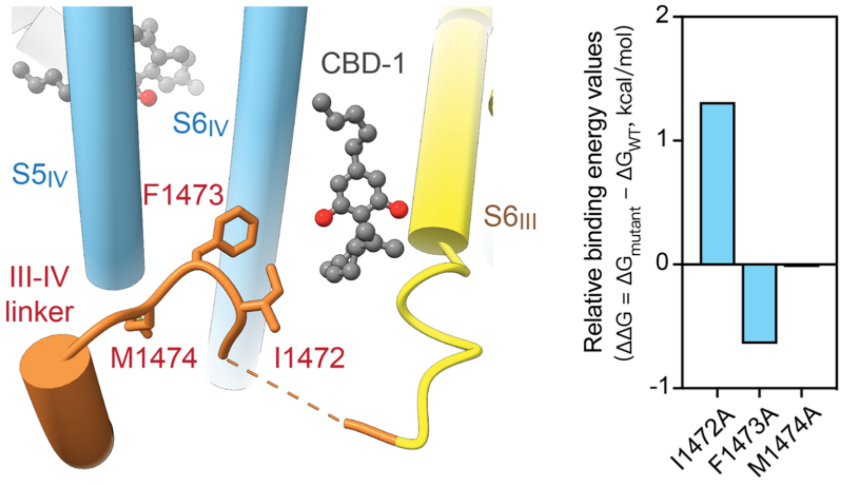

Supplementary Fig. 6 | Position of CBD in the I-site relative to the position of the IFM wedge. *In silico* alanine scanning indicates that Ile1472 might play a role in CBD binding at the I-site. Positive values of relative binding energies, calculated by the Prime-MM/GBSA method, indicate that substituting alanine for the native residue results in less favorable interactions with CBD.

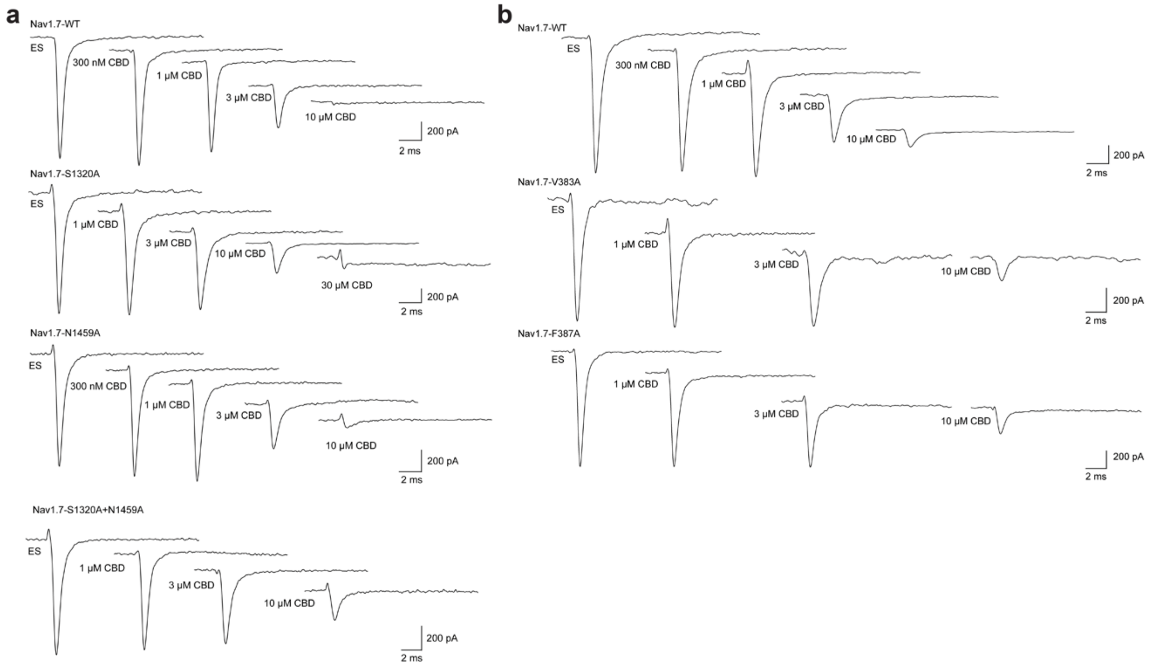

Supplementary Fig. 7 | Blockage of Na$_v$1.7 variants by CBD. Representative traces for blocking the I-site (**a**) and F-site (**b**) mutants of Na$_v$1.7 by CBD at indicated concentrations. The Na$_v$1.7 variants in the first row contain swapped single-point mutations. Please refer to Methods for experimental details and Supplementary Table 1 for the measured parameters.

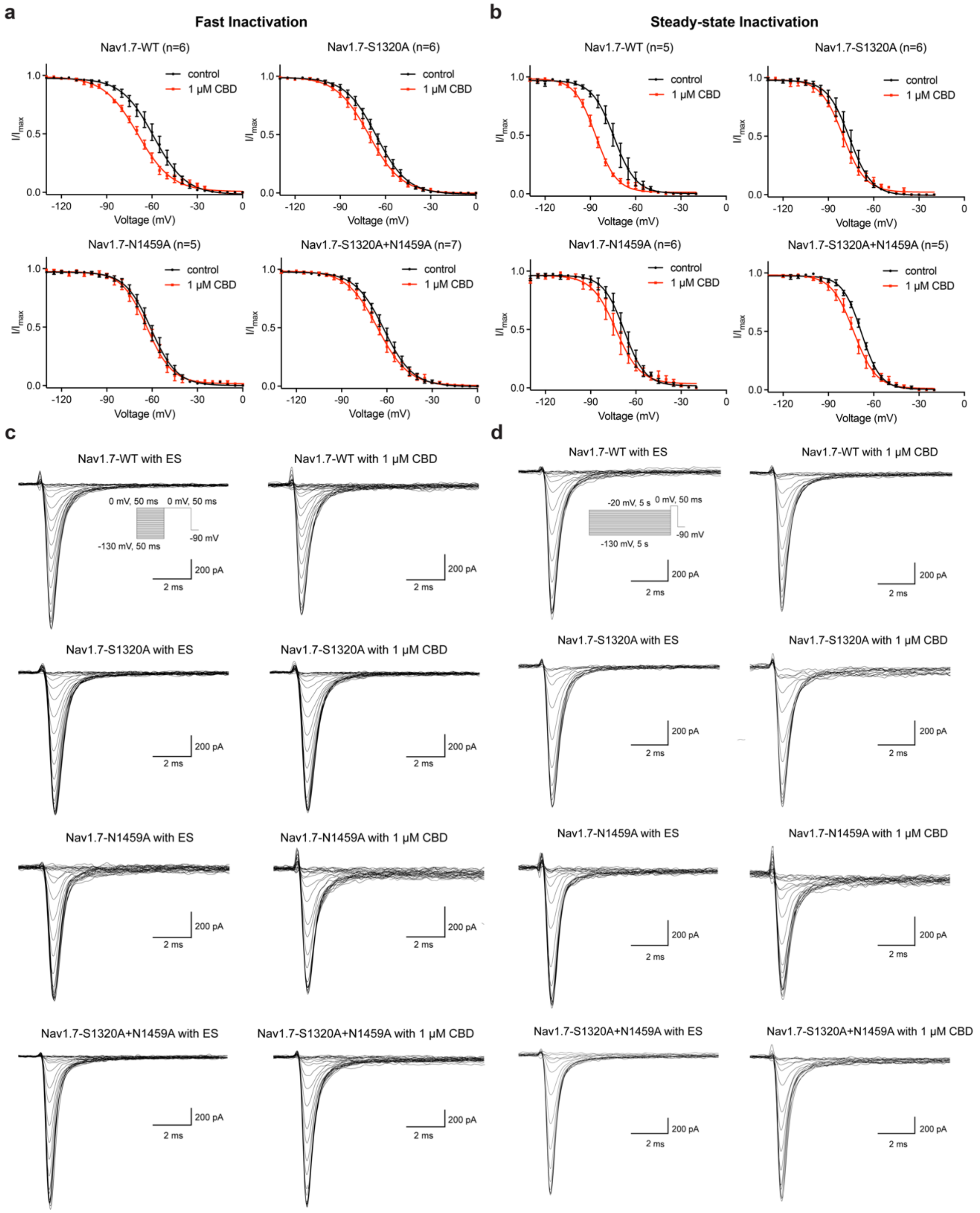

Supplementary Fig. 8 | Fast and steady-state inactivation of Na$_V$1.7 variants by CBD. a,b Voltage-dependent fast (**a**) and steady-state (**b**) inactivation of Na$_V$1.7 and IFM related mutations (S1320A, N1459A, and S1320A+N1459A) after 1 μM CBD treatment. *n* biological independent cells. **c,d** Representative traces for fast (*left*) and steady-state

(*right*) inactivation of $Na_v1.7$ variants after 1 μM CBD treatment. Please refer to Methods section for experimental details and Supplementary Table 2 for the measured parameters.

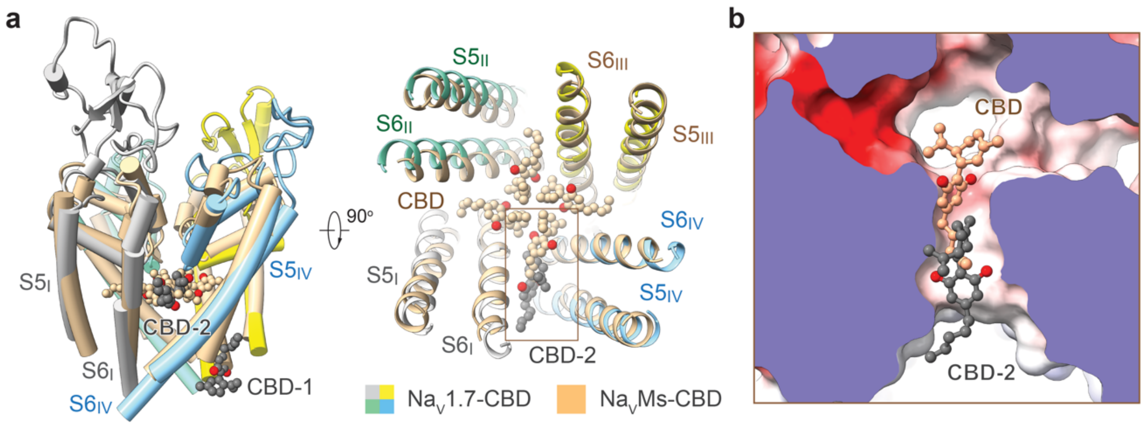

Supplementary Fig. 9 | Comparison of CBD binding in Na_v1.7 and Na_vMs at the F-site. a Deviation of the CBD binding poses in Na_v1.7 and Na_vMs. A side view (*left*) and a top view (*right*) of the superimposed pore domain of CBD-bound Na_v1.7 (domain colored) and Na_vMs (pink, PDB: 6YZ0) are shown. One CBD molecule occupies the fenestration enclosed by repeats I and IV of Na_v1.7, whereas four CBD molecules each binds to a fenestration site in Na_vMs. **b** Different CBD binding poses at the F-site in Na_v1.7 and Na_vMs. The pore domain of Na_v1.7 is shown as a cut-open electrostatic surface.

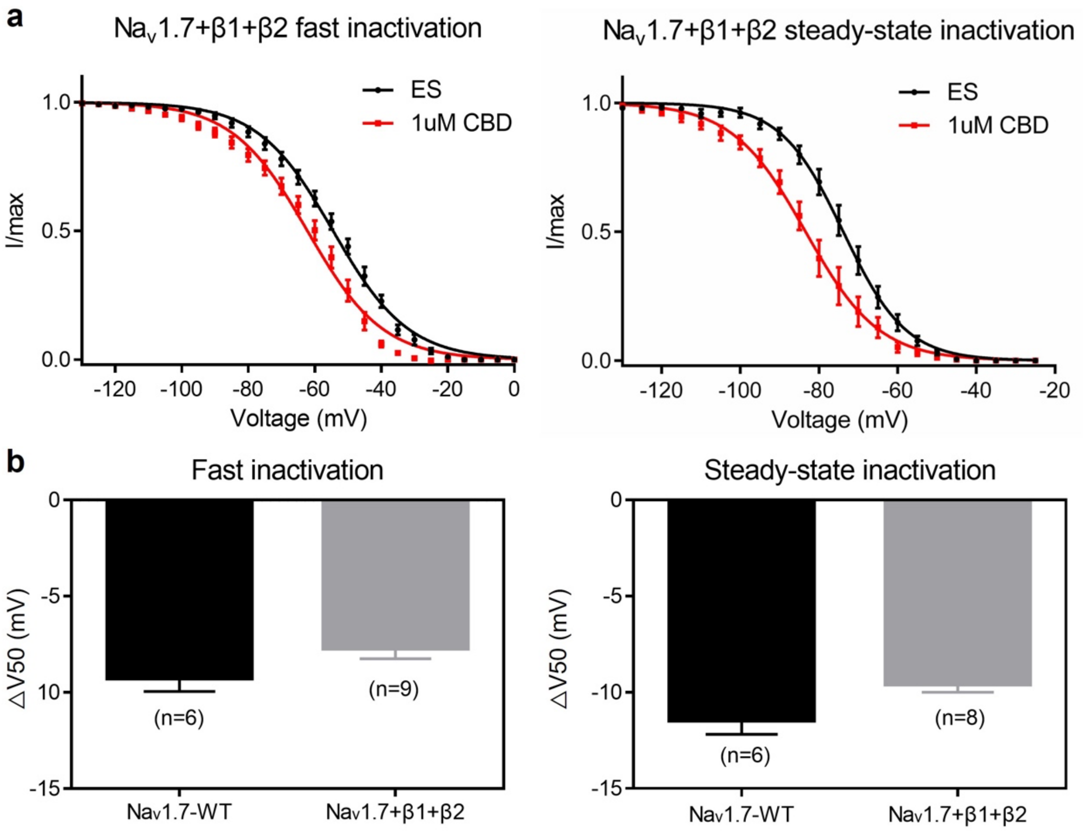

Supplementary Fig. 10 | CBD produces similar effects on the voltage-dependence of channel availability in Na_V1.7 channels studied with or without co-expressed β1 and β2 subunits. a Voltage-dependent fast (*left*) and steady-state (*right*) inactivation of Na_V1.7 and Na_V1.7 co-expressed with β1 and β2 after 1 μM CBD treatment. The voltage-dependence of channel availability was measured for fast inactivation (50-ms prepulses) and steady-state inactivation (5-s prepulses) as described in Methods. **b** Co-expressed with β1 and β2 doesn't modify shifts in fast and steady-state inactivation induced by 1 μM CBD. The ΔV_{50} values for fast inactivation: -9.26 ± 0.69 mV (WT, *n* = 6), -7.71 ± 0.53 mV (*n*= 9); for steady-state inactivation: -11.46 ± 0.72 mV (WT, *n* =6), -9.58 ± 0.42 mV (*n* = 8). Data represent mean ± SEM. *n* biological independent cells.

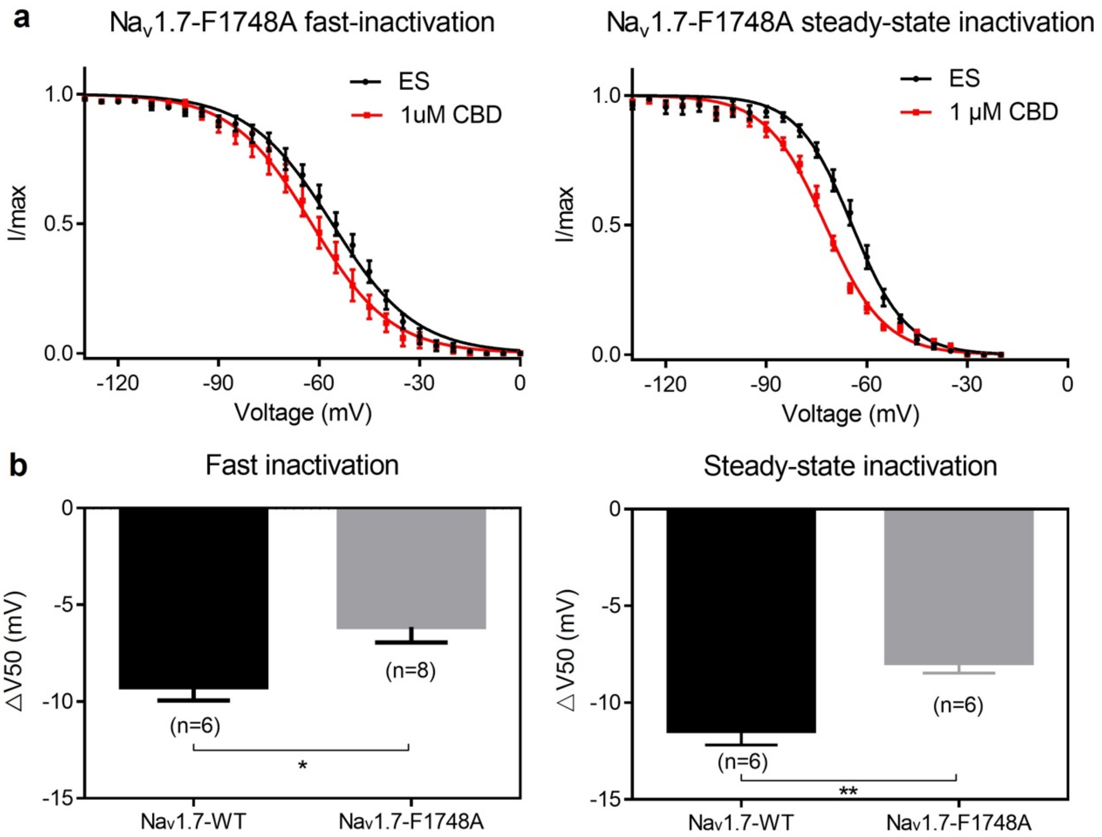

Supplementary Fig. 11 | Effect of mutating F1748 on CBD-induced shift of voltage-dependence of channel availability. a Voltage-dependent fast (*left*) and steady-state (*right*) inactivation of $Na_v1.7$ and $Na_v1.7$-F1748A after 1 µM CBD treatment. The voltage-dependence of channel availability was measured for fast inactivation (50-ms prepulses) and steady-state inactivation (5-s prepulses) as described in Methods. **b** Mutation modify shifts in fast and steady-state inactivation induced by 1 µM CBD. The ΔV_{50} values for fast inactivation: -9.26 ± 0.69 mV (WT, *n* = 6), -6.14 ± 0.81 mV (*n*= 8); for steady-state inactivation: -11.46 ± 0.72 mV (WT, *n* =6), -7.94 ± 0.53 mV (*n* = 6). Data represent mean ± SEM. *n* biological independent cells.

Supplementary Table 1 | Statistics for data collection and structural refinement.

	hNav1.7-CBD
**Data collection and processing**	
Magnification	105,000
Voltage (kV)	300
Electron dose (e-/Å^2)	50
Defocus range (μm)	-1.6~-1.2
Pixel size (Å)	1.114
Symmetry	C1
Initial particle images (no.)	5,126,502
Final particle images (no.)	488,974
Map resolution (Å)	2.8/3.2
FSC threshold (half-map/model-map)	0.143/0.5
**Refinement**	
Initial model used	7W9K
Map sharpening *B* factor (Å^2)	-71.9
Model composition	
Non-hydrogen atoms	13,725
Protein residues	1,574
Ligands	37
B factors (Å^2)	
Protein	128.55
Ligand	134.57
R.m.s deviations	
Bond lengths (Å)	0.003
Bond angles (°)	0.647
Validation	
MolProbity score	1.72
Clashscore	7.99
Poor rotamers (%)	0.77
Ramachandran plot	
Favored (%)	95.83
Allowed (%)	4.17
Disallowed (%)	0.00

Supplementary Table 2 | Concentration-response curves of CBD on Na_v1.7-WT and mutations in HEK293T cells.

		1.7-WT	1.7-V383A	1.7-F387A	1.7-S1320A	1.7-N1459A	1.7-S1320A+N1459A
IC_{50} (μM)		1.82 ± 0.10	3.56 ± 0.58***	3.65 ± 0.78***	3.81 ± 0.42****	2.46 ± 0.28*	4.28 ± 0.67****
P		/	< 0.0001	0.0008	< 0.0001	0.0236	< 0.0001
Slope		1.64 ± 0.15	1.37 ± 0.31	1.24 ± 0.45	1.38 ± 0.22	2.17 ± 0.55	1.27 ± 0.26
P		/	0.3800	0.3246	0.3329	0.2273	0.1904
n	100 nM	1	/	/	/	/	/
	300 nM	5	/	/	/	3	/
	1 μM	12	5	4	3	9	7
	3 μM	8	4	6	5	7	4
	10 μM	7	5	4	5	5	4
	30 μM	/	/	/	2	/	/

* $P < 0.05$ versus WT, *** $P < 0.001$ versus WT, **** $P < 0.0001$ versus WT. Each data point represents mean ± s.e.m and *n* is the number of experimental cells from which recordings were obtained. The extra sum-of-squares F test was used to compare the IC_{50} and slope factor of concentration-response curves. P values for IC_{50} comparatation: < 0.0001, 0.0008, < 0.0001, 0.0236, < 0.0001 (Na_v1.7-WT v.s. Na_v1.7-V383A, Na_v1.7-F387A, Na_v1.7-S1320A, Na_v1.7-N1459A, Na_v1.7-S1320A +N1459A). P values for slope comparatation: 0.3800, 0.3246, 0.3329, 0.2273, 0.1904 (Na_v1.7-WT v.s. Na_v1.7-V383A, Na_v1.7-F387A, Na_v1.7-S1320A, Na_v1.7-N1459A, Na_v1.7-S1320A +N1459A).

Supplementary Table 3 | Fast and steady-state inactivation parameters of $Na_v1.7$ and IFM related mutations in HEK293T cells before and after 1 µM CBD application.

Fast inactivation								
Parameters		$V_{1/2}$ (mV)	P	slope	P	Tau (ms)	P	n
$Na_v1.7$-WT	ES	-60.30 ± 0.58	/	10.84 ± 0.51	/	0.56 ± 0.03	/	6
	1 µM CBD	-69.56 ± 0.38****	< 0.0001	11.74 ± 0.34	0.1549	0.69 ± 0.10	0.2236	6
$Na_v1.7$-S1320A	ES	-66.71 ± 0.39	/	10.28 ± 0.34**	/	0.60 ± 0.05	/	6
	1 µM CBD	-71.17 ± 0.42****	< 0.0001	10.88 ± 0.37	0.2291	0.54 ± 0.02	0.2802	6
$Na_v1.7$-N1459A	ES	-61.54 ± 0.48	/	9.21 ± 0.43	/	0.54 ± 0.03	/	5
	1 µM CBD	-63.63 ± 0.50**	0.0028	9.28 ± 0.44	0.9132	0.54 ± 0.05	0.9519	5
Nav1.7-S1320A+N1459A	ES	-62.78 ± 0.42	/	10.05 ± 0.37	/	0.59 ± 0.05	/	7
	1 µM CBD	-66.36 ± 0.44****	< 0.0001	10.65 ± 0.38	0.2726	0.54 ± 0.04	0.5516	7
**Steady-state inactivation**								
Parameters		$V_{1/2}$ (mV)	P	slope	P	Tau (ms)	P	n
$Na_v1.7$-WT	ES	-75.01 ± 0.64	/	7.93 ± 0.56	/	0.61 ± 0.07	/	6
	1 µM CBD	-86.47 ± 0.33****	< 0.0001	7.51 ± 0.29	0.5224	0.64 ± 0.06	0.7350	6
$Na_v1.7$-S1320A	ES	-76.69 ± 0.59	/	7.66 ± 0.52**	/	0.64 ± 0.07	/	6
	1 µM CBD	-80.08 ± 0.46****	< 0.0001	8.21 ± 0.41	0.4304	0.57 ± 0.04	0.3732	6
$Na_v1.7$-N1459A	ES	-68.20 ± 0.61	/	8.00 ± 0.55	/	0.56 ± 0.09	/	6
	1 µM CBD	-73.22 ± 0.77****	< 0.0001	9.67 ± 0.69	0.0761	0.59 ± 0.06	0.8334	6
Nav1.7-S1320A+N1459A	ES	-68.60 ± 0.26	/	6.83 ± 0.23	/	0.53 ± 0.06	/	5
	1 µM CBD	-73.36 ± 0.45****	< 0.0001	7.99 ± 0.40*	0.0149	0.59 ± 0.06	0.5410	5

* $P < 0.05$ versus WT, *** $P < 0.001$ versus WT, **** $P < 0.0001$ versus WT. Each data point represents mean ± s.e.m and *n* is the number of experimental cells from which recordings were obtained. ES means external solution. The extra sum-of-squares F test was used to compare the $V_{1/2}$ and slope factor of activation fits. P values for $V_{1/2}$ comparatation before and after 1 μM CBD treatment: < 0.0001, < 0.0001, 0.0028, < 0.0001 (fast inactivation); < 0.0001, < 0.0001, < 0.0001, < 0.0001 (steady-state inactivation). P values for slope comparatation before and after 1 μM CBD treatment: 0.1549, 0.2291, 0.9132, 0.2726 (fast inactivation); 0.5224, 0.4304, 0.0761, 0.0149 (steady-state inactivation). P values for tau comparatation before and after 1 μM CBD treatment: 0.2236, 0.2802, 0.9519, 0.5516 (fast inactivation); 0.7350, 0.3732, 0.8334, 0.5410 (steady-state inactivation). Fast inactivation, Na_v1.7-WT, *n* = 6, Na_v1.7-S1320A, *n* = 6, Na_v1.7-N1459A, *n* = 5, Na_v1.7-S1320A +N1459A, *n*=7. Steady-state inactivation, Na_v1.7-WT, *n* = 6, Na_v1.7-S1320A, *n* = 6, Na_v1.7-N1459A, *n* = 6, Na_v1.7-S1320A +N1459A, *n* = 5. Data represent mean ± SEM. *n* biological independent cells.
