# Supplementary material for: Cannabidiol inhibits Nav channels through two distinct binding sites
Source: Nat Commun. 2023 Jun 17;14:3613. doi: 10.1038/s41467-023-39307-6 (PMC10276812; doi:10.1038/s41467-023-39307-6)
Supplement: Supplementary file 3 — Reporting Summary [file 41467_2023_39307_MOESM3_ESM.pdf]

nature portfolio

Corresponding author(s): Nieng Yan
Bruce P. Bean

Last updated by author(s): May 28, 2023

Reporting Summary

Nature Portfolio wishes to improve the reproducibility of the work that we publish. This form provides structure for consistency and transparency in reporting. For further information on Nature Portfolio policies, see our Editorial Policies and the Editorial Policy Checklist.

Statistics

For all statistical analyses, confirm that the following items are present in the figure legend, table legend, main text, or Methods section.

n/a	Confirmed	
☐	☒	The exact sample size (*n*) for each experimental group/condition, given as a discrete number and unit of measurement
☐	☒	A statement on whether measurements were taken from distinct samples or whether the same sample was measured repeatedly
☐	☒	The statistical test(s) used AND whether they are one- or two-sided<br>*Only common tests should be described solely by name; describe more complex techniques in the Methods section.*
☒	☐	A description of all covariates tested
☐	☒	A description of any assumptions or corrections, such as tests of normality and adjustment for multiple comparisons
☐	☒	A full description of the statistical parameters including central tendency (e.g. means) or other basic estimates (e.g. regression coefficient) AND variation (e.g. standard deviation) or associated estimates of uncertainty (e.g. confidence intervals)
☐	☒	For null hypothesis testing, the test statistic (e.g. *F*, *t*, *r*) with confidence intervals, effect sizes, degrees of freedom and *P* value noted<br>*Give P values as exact values whenever suitable.*
☒	☐	For Bayesian analysis, information on the choice of priors and Markov chain Monte Carlo settings
☒	☐	For hierarchical and complex designs, identification of the appropriate level for tests and full reporting of outcomes
☒	☐	Estimates of effect sizes (e.g. Cohen's *d*, Pearson's *r*), indicating how they were calculated

Our web collection on statistics for biologists contains articles on many of the points above.

Software and code

Policy information about availability of computer code

Data collection	All cryo-EM data were collected using SerialEM 3.8.1, and all electrophysiological data were colleced using an EPC10-USB amplifier with Patchmaster software v2*90.2, V2019.
Data analysis	All collected cryo-EM data were processed using cryoSPARC v3.3.2, Relion 4.0.<br>Map and model refinement were processed with UCSF- Chimera v1.14, COOT v0.9.6 and PHENIX v1.21.<br>Electrophysiology data using transiently-transfected cells were acquired using Patchmaster2019 and processed using Origin 2019b.<br>electrophysiology data using the Nav1.7 stable cell line were acquired using pClamp 9 and analyzed using IgorPro 6.1, using DataAccess 9.4 to import data from pClamp to Igor Pro.<br>All figures except Figure 1 were analyzed and prepared using ChimeraX v1.1, Excel 2016 and GraphPad Prism 8.0.2. Figure 1 was analyzed and displayed using Igor Pro 6.1.

For manuscripts utilizing custom algorithms or software that are central to the research but not yet described in published literature, software must be made available to editors and reviewers. We strongly encourage code deposition in a community repository (e.g. GitHub). See the Nature Portfolio guidelines for submitting code & software for further information.

Data

Policy information about availability of data

All manuscripts must include a data availability statement. This statement should provide the following information, where applicable:

- Accession codes, unique identifiers, or web links for publicly available datasets
- A description of any restrictions on data availability
- For clinical datasets or third party data, please ensure that the statement adheres to our policy

The sequences of human Nav1.7, β1 and β2 are available in the following links: Nav1.7 (UniProtID:Q15858): https://www.uniprot.org/uniprotkb/Q15858/entry; β1 (UniProtID:Q07699): https://www.uniprot.org/uniprotkb/Q07699/entry; β2 (UniProtID:O60939): https://www.uniprot.org/uniprotkb/O60939/entry.
The cryo-EM map has been deposited in the Electron Microscopy Data Bank (EMDB) under accession code EMD-29665 [https://www.ebi.ac.uk/pdbe/entry/emdb/EMD-29665] (Nav1.7-CBD). The coordinates have been deposited in the RCSB Protein Data Bank (PDB) under accession code 8G1A [https://doi.org/10.2210/pdb8G1A/pdb] (Nav1.7-CBD).
Previously solved structures mentioned in this study are under the accession codes in PDB: 7W9K [https://doi.org/10.2210/pdb7W9K/pdb] (Nav1.7-apo), 6J8G [https://doi.org/10.2210/pdb6J8G/pdb] (Nav1.7-HWTX IV and STX), 6YZ0 [https://doi.org/10.2210/pdb6YZ0/pdb] (NavMs F208L-CBD), and 6U88 [https://doi.org/10.2210/pdb6U88/pdb] (rTRPV2-CBD), respectively.

Research involving human participants, their data, or biological material

Policy information about studies with human participants or human data. See also policy information about sex, gender (identity/presentation), and sexual orientation and race, ethnicity and racism.

Reporting on sex and gender	N/A
Reporting on race, ethnicity, or other socially relevant groupings	N/A
Population characteristics	N/A
Recruitment	N/A
Ethics oversight	N/A

Note that full information on the approval of the study protocol must also be provided in the manuscript.

Field-specific reporting

Please select the one below that is the best fit for your research. If you are not sure, read the appropriate sections before making your selection.

☒ Life sciences ☐ Behavioural & social sciences ☐ Ecological, evolutionary & environmental sciences

For a reference copy of the document with all sections, see nature.com/documents/nr-reporting-summary-flat.pdf

Life sciences study design

All studies must disclose on these points even when the disclosure is negative.

Sample size	In electrophysiological recordings, more than 6 cells were tested for WT and mutant channels. The number was selected based on previous experience in our lab and other studies for the sample size needed to result in statistically relevant comparisons and was sufficient for performing the statistical tests. The sample size was not applied to the structural analysis.
Data exclusions	For cryo-EM analysis, micrographs with low CTF fitting resolution were excluded, only high resolution and homogeneous particles were retained.
Replication	Replication was not applied to the structural analysis. The final structure of Nav1.7-CBD complex is a represented density of projections from 488,974 individual protein particles. Meanwhile, the gold standard FSC analysis randomly split the dataset into two subsets to avoid over fitting. For electrophysiological recording of both WT and mutant channels, all data have been successfully repeated with at least two batches of samples and all results were similar.
Randomization	In cryo-EM analysis, target particles were selected automatically by software packages. In the electrophysiological experiments, the GFP positive cells were randomly selected for whole-cell patch.
Blinding	All the constructs were recorded and analyzed blindly to avoid bias.

Reporting for specific materials, systems and methods

We require information from authors about some types of materials, experimental systems and methods used in many studies. Here, indicate whether each material, system or method listed is relevant to your study. If you are not sure if a list item applies to your research, read the appropriate section before selecting a response.

Materials & experimental systems

n/a	Involved in the study
☒	☐ Antibodies
☐	☒ Eukaryotic cell lines
☒	☐ Palaeontology and archaeology
☒	☐ Animals and other organisms
☒	☐ Clinical data
☒	☐ Dual use research of concern
☒	☐ Plants

Methods

n/a	Involved in the study
☒	☐ ChIP-seq
☒	☐ Flow cytometry
☒	☐ MRI-based neuroimaging

Eukaryotic cell lines

Policy information about cell lines and Sex and Gender in Research

Cell line source(s)	HEK293F (Invitrogen); HEK293T (Invitrogen); human Nav1.7 HEK cell line (Dr. Sooyeon Jo, Harvard Medical School).
Authentication	No further authentication was performed for commercially available cell lines. Human Nav1.7 cell line was described in PMID: 22442564 and was further authenticated by verifying that voltage-dependent sodium currents were inhibited by the selective Nav1.7 inhibitor PF05089771.
Mycoplasma contamination	Not tested for mycoplasma contamination.
Commonly misidentified lines (See ICLAC register)	No commonly misidentified cell lines were used in this study.
